# The Dual Role of Perivascular Adipose Tissue in Vascular Homeostasis and Atherogenesis: From Physiology to Pathological Implications

**DOI:** 10.3390/ijms26178320

**Published:** 2025-08-27

**Authors:** Raluca Niculescu, Adina Stoian, Emil Marian Arbănași, Eliza Russu, Dragoș-Florin Babă, Andrei Manea, Mircea Stoian, Florina Ioana Gliga, Iuliu Gabriel Cocuz, Adrian Horațiu Sabău, Dan-Alexandru Szabo, Ovidiu Simion Cotoi

**Affiliations:** 1 Department of Pathophysiology, George Emil Palade University of Medicine, Pharmacy, Science and Technology of Târgu Mureș, 540136 Târgu Mureș, Romania; raluca.niculescu@umfst.ro (R.N.); florina.gliga@umfst.ro (F.I.G.); iuliu.cocuz@umfst.ro (I.G.C.); adrian-horatiu.sabau@umfst.ro (A.H.S.); ovidiu.cotoi@umfst.ro (O.S.C.); 2 Doctoral School of Medicine and Pharmacy, George Emil Palade University of Medicine, Pharmacy, Science and Technology of Târgu Mureș, 540139 Târgu Mureș, Romania; emil.arbanasi@umfst.ro (E.M.A.); andrei.manea@umfst.ro (A.M.); 3 Department of Vascular Surgery, George Emil Palade University of Medicine, Pharmacy, Science and Technology of Târgu Mureș, 540139 Târgu Mureș, Romania; eliza.russu@umfst.ro; 4 Regenerative Medicine Laboratory, Centre for Advanced Medical and Pharmaceutical Research (CCAMF), George Emil Palade University of Medicine, Pharmacy, Science and Technology of Târgu Mureș, 540139 Târgu Mureș, Romania; 5 Department of Cell and Molecular Biology, George Emil Palade University of Medicine, Pharmacy, Science and Technology of Târgu Mureș, 540142 Târgu Mureș, Romania; dragos-florin.baba@umfst.ro; 6 Department of Radiology, George Emil Palade University of Medicine, Pharmacy, Science and Technology of Târgu Mureș, 540139 Târgu Mureș, Romania; 7 Department of Anesthesiology and Intensive Care Medicine, George Emil Palade University of Medicine, Pharmacy, Science and Technology of Târgu Mureș, 540103 Târgu Mureș, Romania; mircea.stoian@umfst.ro; 8 Department M2, Faculty of Medicine, George Emil Palade University of Medicine, Pharmacy, Science and Technology of Târgu Mureș, 38 Gheorghe Marinescu Street, 540139 Târgu Mureș, Romania; dan-alexandru.szabo@umfst.ro

**Keywords:** perivascular adipose tissue, atherosclerosis, adiponectin, adipokines, leptin, cytokines

## Abstract

Atherosclerosis is now recognized as a chronic inflammatory disease of the arterial wall, in which perivascular adipose tissue (PVAT) has evolved from a passive structural component to a key player in regulating vascular homeostasis and the pathophysiology of atherosclerosis, playing an active, not just structural, role. PVAT surrounds blood vessels and influences them metabolically, immunologically, and vascularly by secreting adipokines, cytokines, and other bioactive mediators. Under physiological conditions, PVAT has protective roles, as it produces adiponectin, nitric oxide (NO), and other vasodilatory factors that help maintain vascular tone and reduce inflammation. In particular, brown-like PVAT (rich in Uncoupling Protein-1 (UCP1) and mitochondria) offers significant vasoprotective effects. Under pathological conditions (obesity, dyslipidemia, insulin resistance), PVAT undergoes a phenotypic transition towards a pro-inflammatory profile by increasing leptin, tumor necrosis factor-alpha (TNF-α), and interleukin-6 (IL-6) secretion and decreasing adiponectin, contributing to endothelial dysfunction, vascular smooth muscle cell (VSMC) proliferation, local immune cell recruitment, extracellular matrix (ECM) remodeling, and fibrosis. PVAT plays a complex role in vascular health and disease, interacting with systemic metabolism through the secretion of bioactive molecules. Metabolic imbalances can promote PVAT inflammation. Epigenetic alterations and micro ribonucleic acid (miRNAs) can influence PVAT inflammation, and modern imaging methods for PVAT assessment, such as the fat attenuation index (FAI) and artificial intelligence-assisted radiomic profiling, may become predictive biomarkers of cardiac risk. Future directions aim to identify biomarkers and develop targeted therapies that modulate PVAT inflammation and dysfunction in the context of cardiovascular diseases.

## 1. Introduction

The concept that atherosclerosis is a chronic inflammatory disease of the arterial wall gained traction in the 1980s when studies revealed elevated levels of acute-phase proteins, such as C-reactive protein (CRP), in patients with coronary atherosclerotic disease and myocardial ischemia [1]. Subsequently, elevated CRP levels were shown to be a predictor of cardiovascular disease events [2].

The accumulation of lipids, inflammatory cells, and fibrous tissue within the intimal layer of large- and medium-sized arteries is a characteristic of atherosclerosis, which is the leading cause of cardiovascular diseases worldwide, accounting for approximately one-third of adult deaths [3]. Although initially considered a straightforward lipid storage disorder, it is now recognized as an active and complex process influenced by inflammatory, hemodynamic, and metabolic factors [4].

PVAT surrounds blood vessels, plays a vasoprotective role, and serves important metabolic functions that influence cardiovascular health [5]. Furthermore, recent studies suggest that chronic PVAT inflammation is associated with vascular stiffening, collagen accumulation, and the activation of pro-atherogenic pathways mediated by NF-κB (nuclear factor kappa-light-chain-enhancer of activated B cells) and the NLRP3 (NOD-, LRR-, and pyrin domain-containing protein 3) inflammasome, which contributes to plaque instability. These findings support the hypothesis that PVAT may be a valuable therapeutic target in cardiovascular disease prevention [6].

This narrative review aims to examine the recent literature on the role of PVAT in atherogenesis, focusing on the molecular mechanisms involved and phenotypic changes of adipose tissue in various pathological contexts. Additionally, modern imaging and histological evaluation methods for PVAT, along with their clinical implications, are discussed. 

## 2. Anatomy and Physiological Role of PVAT

### 2.1. Anatomical Distribution and Structural Differences of PVAT

During embryogenesis, adipocytes and blood vessels develop in close proximity, with early adipocytes emerging from mesenchymal perivascular cells or pericytes embedded around arterioles. Numerous studies suggest a shared progenitor origin for adipocytes and endothelial cells, indicating that the differentiation pathway may rely on local environmental signals, such as those that influence the expression of alpha-smooth muscle actin (α-SMA) in perivascular stem cells. This close relationship between adipogenesis and angiogenesis persists into adulthood, facilitating both the growth of adipose tissue, as observed in obesity, and the preservation of adipocyte counts under normal physiological conditions [7].

PVAT is distributed widely throughout the vasculature, surrounding large elastic arteries, such as the aorta, as well as muscular arteries, including coronary, mesenteric, and renal arteries, and even small resistance vessels. The composition and function of PVAT vary significantly depending on the anatomical site. This heterogeneity is evident in differences in cellular content, vascularization, innervation, and the profile of bioactive substances released [8].

**Figure 1:**
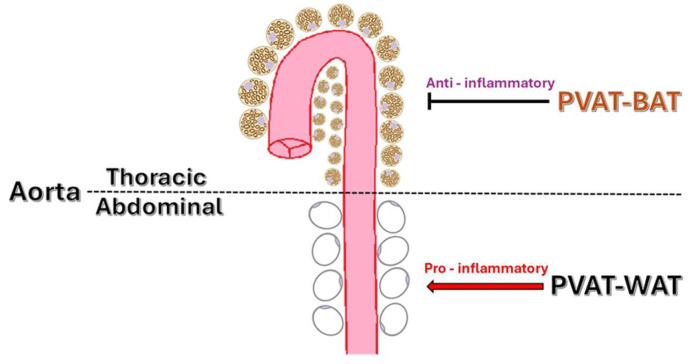
Regional distribution of PVAT in the aorta.

The phenotypic diversity of PVAT includes white, brown, and beige adipose characteristics, which influence its function. In some regions, PVAT shares features with brown adipose tissue (BAT), including generating thermogenic effects during cold exposure or adrenergic stimulation. Brown-like PVAT, which is primarily located in the thoracic aorta regions, is rich in UCP-1 and mitochondria and is associated with increased energy expenditure and vasoprotective effects. BAT uses lipids to generate heat by uncoupling oxidation in the mitochondrial electron transport chain [9,10]. This process helps remove lipids from the bloodstream, preventing their accumulation in white adipose tissue (WAT) and other organs. White-like PVAT, such as that surrounding the abdominal aorta and mesenteric vessels, tends to produce more pro-inflammatory cytokines and is linked to greater oxidative stress in pathological states compared to BAT-like (Figure 1). Excessive lipid accumulation in WAT leads to adipocyte hypertrophy and dysfunction, resulting in increased secretion of harmful adipokines and pro-inflammatory cytokines. These substances impair vascular endothelial function [10].

**Figure 2:**
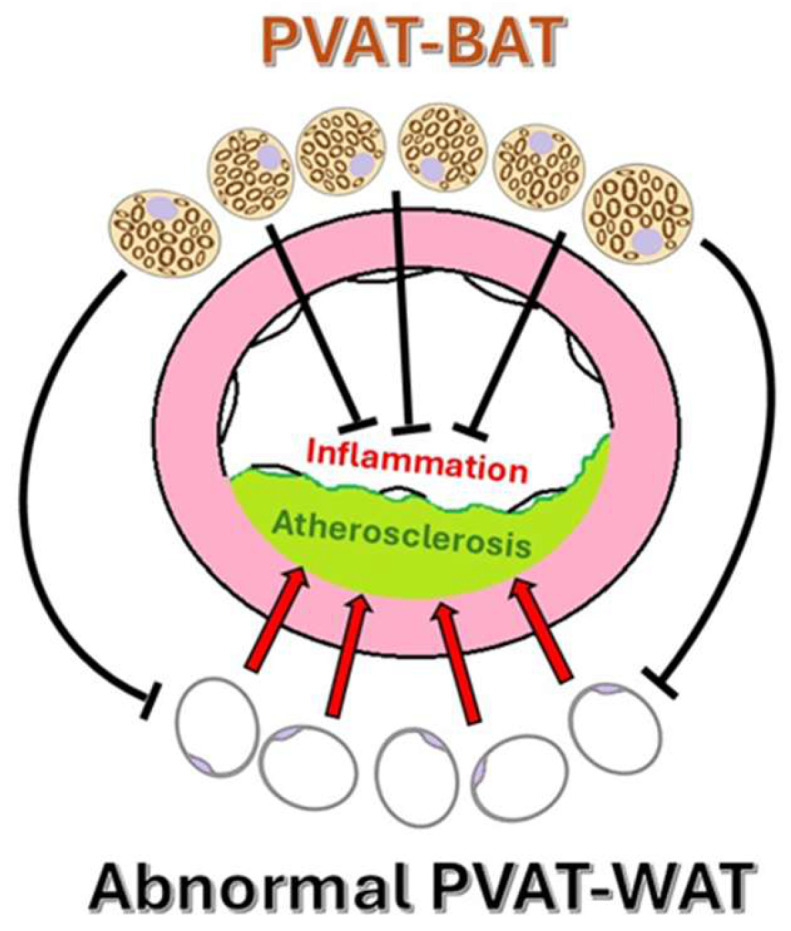
PVAT Plasticity: Atherogenic potential of WAT vs. vascular protection by BAT.

Under normal physiological conditions, PVAT prevents atherogenesis by sequestering fatty acids and possessing thermogenic capabilities [10,11]. Dysfunction in WAT is linked to the development of atherosclerosis, while activation of BAT appears to have a protective effect, indicating plasticity that can be harnessed for therapeutic benefit (Figure 2). This hypothesis is supported by studies that demonstrate a reduction in hypercholesterolemia and atherosclerosis in hyperlipidemic mice treated with β3-adrenergic receptor agonists [10].

Coronary PVAT is particularly interesting because of its anatomical proximity to the heart and its direct involvement in the function of the coronary arteries. Unlike other PVAT depots, coronary PVAT shares microcirculation with the coronary arteries and lacks a clear fascial boundary, allowing for direct diffusion of bioactive molecules. Structurally, it exhibits a mixed adipocyte phenotype, containing both white and beige adipocytes, and is highly vascularized and innervated [12].

In smaller arteries, such as the mesenteric and renal arteries, PVAT is thinner and less organized, often integrated into the adventitia rather than forming a distinct external layer. Despite its relatively small volume, mesenteric PVAT exerts significant vasoactive effects, possibly due to its proximity to the vascular wall and high sensitivity to metabolic cues [13]. This anatomical variation suggests region-specific functional roles, with thoracic PVAT potentially providing more anti-contractile and protective effects compared to the more inflammatory profile of abdominal PVAT [14,15].

### 2.2. Normal Paracrine Functions and Physiological Roles of PVAT

PVAT was long overlooked in cardiovascular research and was traditionally viewed as a mere structural component. However, adipose tissue, including perivascular depots, acts not only as an energy reservoir but also as a dynamic endocrine organ capable of interorgan communication [16]. Over the past decade, studies have revealed that PVAT has a central physiological role and regulates vascular function through the secretion of adipokines (bioactive peptides with autocrine, paracrine, and endocrine effects), cytokines, gasotransmitters, and other bioactive molecules [17]. Through the release of these adipokines, adipose tissue influences a wide range of distant organs such as the heart, brain, liver, skeletal muscle, and vascular system. These adipokines can exert both pro-inflammatory and anti-inflammatory effects, and the balance between them plays a pivotal role in maintaining systemic and vascular homeostasis [16]. Under physiological conditions, PVAT helps maintain vascular tone and inhibits inflammation through its protective actions on the endothelium and VSMCs [18].

Ling Chang et al. used a murine model with a genetic deficiency of peroxisome proliferator-activated receptor-γ in smooth muscle cells (SMPG KO mice), in which PVAT did not form around blood vessels. This likely results from the receptor’s deletion also affecting perivascular adipocyte precursor cells. The authors noted that, under these conditions, cold exposure—due to the absence of PVAT thermogenic activity—led to endothelial dysfunction and increased heat loss, which did not happen in mice with normal PVAT. In healthy mice, cold exposure stimulated thermogenesis through efficient lipid clearance, improving endothelial function and reducing the progression of atherosclerosis. Therefore, the authors concluded that because PVAT shares characteristics with BAT in certain aspects, it acts as a vasoactive organ involved in cold adaptation and intravascular thermoregulation, also offering a protective effect against the development of atherosclerosis [19].

PVAT has a dual role, affecting both vascular physiology and pathophysiology. On one hand, after vascular injuries, PVAT undergoes inflammatory changes. It appears to promote inflammatory lesions in the vascular wall, contributing to the development of atherosclerosis [19], which suggests an association between PVAT phenotypic changes and vascular diseases. Newly developed computerized tomography-based methods also emphasize this connection [20]. On the other hand, exposure to cold or β3-adrenergic receptor (β3AR) agonists causes white-like PVAT to develop a brown adipocyte-like phenotype through the action of transcriptional regulatory factors, including PRDM16 [21,22]. This phenomenon is called browning or “beiging,” observed in both rodent models and humans, and contributes to energy homeostasis [23,24]. In murine models of endovascular injury, this is followed by the accumulation of macrophages in PVAT, along with its transition to the beige phenotype characterized by reduced inflammation and pathological vascular effect remodeling. Beige adipocytes abundantly express neuregulin 4 (Nrg4), which promotes the alternative activation of macrophages, thereby supporting the resolution of vascular inflammation. Yusuke Adachi et al. showed that genetic blockade of PRDM16 expression worsens posttraumatic inflammation and vascular remodeling by preventing the transition to the beige phenotype [25]. At the same time, in murine models and human small arteries, PVAT presence reduces the response to vasoconstrictor agents [19,26,27]. Recent evidence underscores the vasoprotective role of nicotinamide adenine dinucleotide (NADPH) oxidase 4 (NOX4) in resistance arteries, primarily through the generation of hydrogen peroxide, which supports endothelial function and vascular relaxation [28]. The absence of NOX4 exacerbates vascular dysfunction, an effect further intensified by the presence of PVAT, suggesting a harmful synergism between PVAT and NOX4 deficiency [29]. These findings underscore the significance of NOX4 in maintaining vascular homeostasis and suggest that PVAT plays a role in vascular impairment under pathological conditions.

### 2.3. Physiological Vascular Tone Modulation by PVAT

PVAT exerts a paracrine anti-contractile effect on adjacent vessels under physiological conditions. This effect is mediated through the release of relaxing factors collectively termed “adipocyte-derived relaxing factors”, such as hydrogen sulfide (H_2_S), NO), angiotensin 1–7 (Ang1–7), and adiponectin, which prevent lipid deposition and inhibit VSMC proliferation to promote relaxation and decrease vascular resistance [30]. In the thoracic aorta, the anti-contractile function of PVAT is particularly well defined. The release of NO and H_2_S is facilitated by enzymes such as endothelial nitric oxide synthase (eNOS) and cystathionine γ-lyase, both of which are expressed in PVAT adipocytes. Additionally, PVAT modulates the sympathetic nervous input through local innervation, contributing to the fine-tuning of vascular reactivity [31].

Adiponectin enhances endothelial NO production, inhibits VSMC proliferation, and reduces oxidative stress. It exerts anti-inflammatory effects and improves insulin sensitivity [17]. At physiological levels, leptin supports endothelial function and angiogenesis. It induces endothelium-dependent vasorelaxation through the stimulation of eNOS and NO synthesis [32,33]. Plasma concentrations of omentin are reduced in overweight and obese individuals but increase after weight loss, suggesting a beneficial role in metabolic regulation. Experimental studies have demonstrated that omentin induces endothelium-dependent vasorelaxation through NO production, which may aid in blood pressure regulation. Additionally, omentin has been shown to suppress TNF-α-induced inflammatory responses in human vascular endothelial cells, highlighting its anti-inflammatory properties [34]. The main adipokines are illustrated in Table 1.

Through this adipokine profile, PVAT serves as a regulatory structure that influences vascular homeostasis, metabolic integration, and immune responses. Notably, in physiological states, the balance of secreted adipokines skews toward anti-inflammatory and vasoprotective profiles, thereby contributing to the maintenance of normal vascular tone and structural integrity [32].

## 3. SAT, VAT, and PVAT Characteristics

PVAT is defined as the adipose tissue located within a radial distance equal to the luminal diameter of the adjacent artery, up to a maximum of 2 cm for vessels with diameters greater than 2 cm, such as the aorta. PVAT shares anatomical continuity with surrounding fat depots, such as epicardial adipose tissue (EAT) in the case of coronary arteries but exhibits distinct biological functions due to its proximity to the vascular wall [43,44,45]. Subcutaneous adipose tissue (SAT) refers to the white adipose tissue within the reticular dermis of the skin. It constitutes the largest adipose depot in the human body and exhibits a sex-specific distribution, being predominantly found in the trunk in males and around the hips and lower limbs in females. Abdominal SAT is further classified into superficial and deep layers, with the latter exhibiting phenotypic similarities to visceral adipose tissue [44,46]. Visceral adipose tissue (VAT) comprises fat depots within the thoracic and abdominal cavities and surrounding internal organs. Abdominal VAT includes mesenteric, peritoneal, and retroperitoneal (perirenal) components, while thoracic VAT comprises EAT, pericardial adipose tissue, and other non-pericardial thoracic fat depots. VAT is closely associated with metabolic and cardiovascular diseases because of its high inflammatory potential [44,46].

To better understand the specific role of PVAT in vascular physiology and pathology, it is essential to compare its structural and functional characteristics with those of other adipose depots, such as SAT and VAT. Table 2 offers an overview of SAT, VAT, and PVAT features, highlighting key differences in cellular composition, endocrine function, inflammatory potential, thermogenic capacity, and their respective implications for metabolic and cardiovascular health [11,45,47,48,49].

## 4. PVAT in Pathological States

### 4.1. Cellular Composition of PVAT and Crosstalk with the Systemic Metabolism 

In its normal state, PVAT contains various cells, including adipocytes, macrophages, T lymphocytes, and mast cells. These cells work together to maintain an anti-inflammatory environment, which is essential for healthy vascular function [51].

PVAT should be viewed not only as a repository of fat but also as an active endocrine organ. It secretes bioactive molecules that influence nearby blood vessels and regulate vascular tone or are released into the bloodstream to other parts of the body, thereby exerting a significant impact on the systemic metabolism. Pro-inflammatory adipokines, which are signaling molecules released by adipose tissue, play a crucial role in promoting vascular inflammation. This inflammation triggers the recruitment and infiltration of immune cells into the vascular wall. This process exacerbates the proliferation and migration of SMCs and contributes to the buildup of fat and the development of atherosclerotic plaques. As these SMCs multiply and migrate, they contribute to structural changes within the vessel wall, leading to significant vascular dysfunction [10]. By secreting molecules involved in metabolism, PVAT contributes to systemic metabolic dysfunction, including obesity, insulin resistance, and dyslipidemia [43].

**Figure 3:**
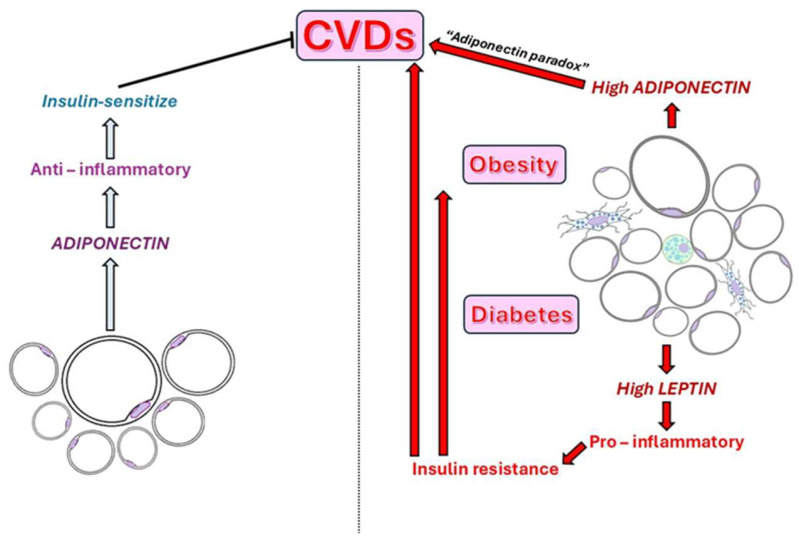
“Adiponectin paradox”.

In obesity, PVAT shifts from a vasoprotective to a pro-inflammatory phenotype, characterized by reduced NO availability, increased oxidative stress, and heightened secretion of pro-inflammatory cytokines, such as leptin, TNF-α, and IL-6, while the secretion of anti-inflammatory adipokines, such as adiponectin, is reduced [10,60,61]. Low levels of adiponectin are associated with obesity, diabetes, hypertension, and various cardiovascular diseases, contributing to insulin resistance and diminished glucose utilization in peripheral tissues [62]. Conversely, elevated levels of adiponectin can also lead to adverse effects, as they have been linked to certain cardiovascular diseases, a phenomenon referred to as the “adiponectin paradox” (Figure 3) [63]. This imbalance favors the recruitment and activation of immune cells (monocytes, macrophages, and dendritic cells) into the PVAT, thereby intensifying inflammation. Macrophages, especially M1-type macrophages, play a central role in PVAT inflammation, secreting pro-inflammatory cytokines, reactive oxygen species (ROS), and matrix metalloproteinases (MMPs) [43,64]. These changes drive endothelial dysfunction, VSMC proliferation, and the remodeling of the ECM, leading to increased production of ROS, particularly superoxide and hydrogen peroxide [65,66]. This dysregulation of adipokine secretion from PVAT contributes to the development of systemic insulin resistance, a hallmark of type 2 diabetes [67]. Furthermore, insulin resistance within PVAT exacerbates adipocyte dysfunction and local inflammation [43,68]. Insulin resistance causes disorders in lipid metabolism, also affecting the lipid composition of PVAT [69]. The bidirectional interaction maintains a vicious cycle in which metabolic imbalance promotes PVAT inflammation, which, in turn, exacerbates vascular injury [57]. Body mass index (BMI), a surrogate for general adiposity, is closely linked to the expansion of PVAT and inflammatory changes, particularly in the coronary circulation [70].

Bartuskova et al. [71] showed that the presence of CD14+ CD16+ CD36^high^ macrophages in visceral adipose tissue correlated with cardiovascular risk factors, and CD14+ CD16+ CD36^low^ macrophages increased after statin treatment and were associated with reduced cardiovascular risk factors. They also reported that the levels of the CD14+ CD16+ CD36^high^ macrophage subtype in PVAT underline how inflammation of perivascular adipose tissue has a direct impact on the arterial wall from the early stages of atherosclerosis, highlighting the importance of local monitoring of this compartment in the assessment of cardiovascular risk [71].

Additionally, epigenetic alterations in PVAT may underlie obesity- and diabetes-related vascular dysfunction via disrupted mitochondrial activity and increased inflammation. For example, epigenetic alterations leading to the repression of peroxisome proliferator-activated receptor gamma coactivator 1α (PGC-1α) alter the expression of UCP1 and peroxisome proliferator-activated receptor gamma (PPARγ) in the epicardial adipose tissue of rats fed a high-fat diet. Reduced expression of PGC-1α and UCP1 in epicardial adipose tissue is linked to epigenetic suppression of these genes in PVAT [72]. Therefore, the relationship between BMI, systemic metabolism, and PVAT characteristics provides valuable insight into cardiovascular risk beyond traditional metrics [73].

### 4.2. Adipokines and Cytokines Secreted by Dysfunctional PVAT

Dysfunctional PVAT serves as a source of pro-inflammatory adipokines and cytokines that negatively affect vascular function. Leptin exhibits a context-dependent duality in its vascular effects, depending on the physiological or pathological context. Normally, it has vasoprotective effects. However, in pathological conditions—such as obesity or metabolic syndrome—elevated leptin levels become pro-inflammatory, increasing the production of pro-inflammatory cytokines, such as TNF-α and IL-6, exacerbating vascular inflammation and contributing to the development of atherosclerosis [10,74]. TNF-α is a crucial pro-inflammatory cytokine that increases the expression of cell adhesion molecules, such as intercellular adhesion molecule-1 (ICAM-1) and vascular cell adhesion molecule-1 (VCAM-1) (), promoting leukocyte infiltration into the vascular wall and contributing to endothelial dysfunction [10]. IL-6 has a dual role, participating in both the inflammatory response and metabolic regulation. In the context of dysfunctional PVAT, IL-6 contributes to the development of oxidative stress and endothelial dysfunction [10].

Initially identified as a factor associated with insulin resistance, resistin also exhibits pro-inflammatory properties. Resistin is associated with various biological processes, including the proliferation of endothelial cells, angiogenesis, the expression of VCAM-1 and ICAM-1 in endothelial cells, and the generation of ROS, which contribute to endothelial dysfunction. Resistin facilitates macrophage recruitment, stimulating the production of cytokines, such as TNF-α and IL-6 [10].

Understanding the molecular mechanisms underlying this phenotypic transition of PVAT presents opportunities for developing therapeutic strategies that aim to reduce perivascular inflammation and alleviate associated vascular dysfunction [75]. The main inflammatory markers that induce endothelial dysfunction are illustrated in Table 3.

### 4.3. Atherosclerosis and Pathophysiological Mechanisms Linking PVAT to Atherosclerosis

Atherosclerosis is characterized by the formation of fibrofatty lesions in the vascular wall, where persistent inflammation is the main factor leading to these changes [85]. The process may develop asymptomatically in the first decades with the buildup of cholesterol-carrying low-density lipoprotein (LDL) particles in the arterial walls [86]. As lipoproteins gather in the subendothelial space, endothelial cells begin to express adhesion molecules, such as VCAM-1 and ICAM-1, which lead to the recruitment of monocytes and other leukocytes. Monocytes differentiate into macrophages under the influence of granulocyte-macrophage colony-stimulating factor (GM-CSF) and macrophage colony-stimulating factor (M-CSF) produced by endothelial cells and other cell types [87,88].

By engulfing lipoproteins, macrophages are transformed into foam cells, which are trapped in the arterial intima. They eventually die, forming a core area that contains apoptotic and necrotic cells, as well as cholesterol crystals [88]. Interferon gamma (IFN-ƴ) and lipopolysaccharides activate the inflammatory subsets of M1 macrophages (type 1). Oxidized phospholipids can induce a distinct macrophage phenotype, different from M1 (pro-inflammatory) and M2 (type 2, anti-inflammatory) cells, termed Mox cells, suggesting a dynamic process at the arterial wall level and not just a simplistic classification of macrophages; these cells are engaged in crosstalk with other cells, especially T lymphocytes [88,89].

Fatty streaks develop into fibrofatty lesions and subsequently into atheromatous plaques with a necrotic core surrounded by a fibrous cap. Vulnerable plaques have a thin fibrous cap and a large necrotic core with an active inflammatory process [90]. The rupture of these plaques is responsible for thrombotic events. A recently published study of ours highlighted correlations between EAT thickness and the presence of unstable atherosclerotic plaques at the level of the left anterior descending artery and left circumflex artery [91].

PVAT has emerged as a critical player in vascular health, particularly because of its close anatomical and functional relationship with the vascular wall. Under physiological conditions, PVAT exerts anti-inflammatory and vasorelaxant effects, thereby supporting vascular homeostasis. In conditions such as obesity, aging, insulin resistance, dyslipidemia, or chronic oxidative stress, PVAT undergoes phenotypic switching, adopting a pro-inflammatory profile leading to increased secretion of leptin, IL-6, and TNF-α, while decreasing adiponectin levels. This promotes endothelial dysfunction, vascular remodeling, and the formation of atherosclerotic plaques. Thus, PVAT is not just a passive bystander but an active participant in the initiation and progression of atherosclerosis [7,92]. This section explores four interrelated pathophysiological mechanisms through which PVAT drives vascular dysfunction and atherogenesis.

#### 4.3.1. Endothelial Dysfunction

The endothelium is the primary vascular layer exposed to PVAT-derived signaling molecules. In healthy conditions, PVAT enhances NO synthesis through the actions of adiponectin. By releasing vasoactive molecules with vasodilatory effects, such as NO, H_2_S, leptin, and adiponectin, PVAT has a protective effect on blood vessels. Under pathological circumstances, PVAT shifts to a pro-inflammatory and pro-oxidative state, secreting cytokines like TNF-α, IL-6, and leptin, which reduce NO bioavailability and increase the production of ROS [93]. The beneficial effect is diminished in cases of obesity, leading to the formation of an “obesity triangle” characterized by inflammation, hypoxia, and oxidative stress at the PVAT level. Leptin is elevated in inflamed PVAT and promotes oxidative stress, vascular smooth muscle proliferation, and endothelial activation. Higher circulating levels of leptin are linked to increased PVAT density and plaque burden in imaging studies [94,95].

**Figure 4:**
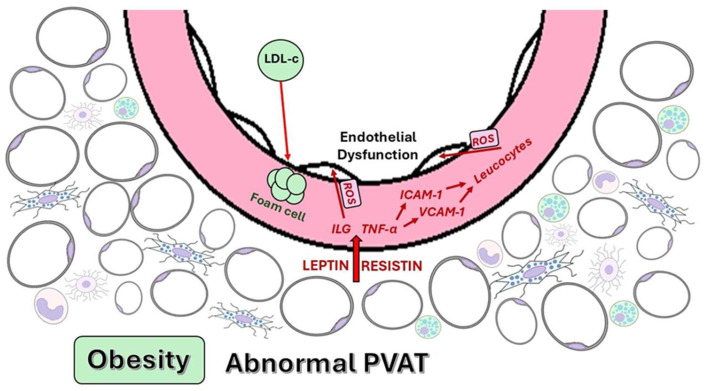
PVAT-mediated endothelial dysfunction.

The NO/ROS imbalance leads to impaired endothelium-dependent vasodilation, increased permeability, and the upregulation of adhesion molecules, such as VCAM-1 and ICAM-1, which favor monocyte adhesion and migration into the intima. The oxidative stress generated within PVAT also inhibits eNOS activity and promotes eNOS uncoupling, thereby worsening vascular dysfunction (Figure 4) [96,97].

Adiponectin, an anti-inflammatory adipokine, is generally reduced in individuals with high PVAT inflammation. Lower levels of adiponectin are associated with endothelial dysfunction and increased cardiovascular risk [98]. In cases of high-fat diet-induced obesity, reduced adiponectin levels from PVAT contribute to endothelial dysfunction, which can be reversed by restoring adiponectin levels [99]. Studies involving genetically modified mice that lack adiponectin or overexpress TNF-α in adipose tissue have demonstrated accelerated endothelial dysfunction and plaque formation, underscoring the pivotal role of PVAT in modulating endothelial health [97].

A study on Sprague–Dawley rats explored the effects of mangiferin, a xanthone glucoside derived from *Anemarrhena asphodeloides* Bunge that is widely used in traditional Chinese medicine for treating diabetes mellitus. Mangiferin enhanced the release of exosomes—nanometric extracellular vesicles involved in intercellular communication and composed of proteins, miRNAs, and non-coding RNAs—under inflammatory conditions from PVAT. These exosomes were taken up by endothelial cells, leading to a reduction in apoptosis, increased cell migration, promotion of vasodilation, decreased expression of inflammatory cytokines, and increased NO production. These beneficial effects are mediated through the inhibition of NF-kB signaling [100]. Therefore, PVAT could serve as a therapeutic target for alleviating endothelial dysfunction.

#### 4.3.2. VSMC Modulation

PVAT significantly influences the growth and migration of VSMCs, processes that are crucial in proliferative vascular diseases, such as atherosclerosis, restenosis, and hypertension. PVAT releases various growth factors and inhibitors, including visfatin and adiponectin, which have direct effects on the proliferation of VSMCs. Visfatin, an adipokine secreted by PVAT, promotes VSMC proliferation and exhibits anti-apoptotic properties. In contrast, adiponectin plays an anti-inflammatory and antiatherogenic role by inhibiting VSMC proliferation and protecting against neointimal hyperplasia following vascular injury. The local renin–angiotensin system in PVAT generates angiotensin II, which has proliferative effects, and angiotensin 1–7, which acts as a vasodilator, thus influencing vascular tone and potential VSMC growth [101].

PVAT-derived pro-inflammatory cytokines also influence the behavior of VSMCs, promoting their migration from the tunica media into the intima, as well as proliferation and ECM production, which are crucial for neointimal hyperplasia and plaque development [82]. Adipokines produced by PVAT, such as leptin, visfatin, lipocalin-2 (LCN-2), and fatty acid-binding protein 4 (FABP4), promote the proliferation of VSMCs and activate macrophages through the p38 MAPK (mitogen-activated protein kinase) signaling pathway, PKC-β (protein kinase C beta) activation, and ERK (extracellular signal-regulated kinase) 1/2-dependent pathways, reinforcing the pro-atherogenic role of this adipose compartment and increasing the thickness and stiffness of the vascular wall [10]. Moreover, leptin enhances VSMC migration by activating MMPs, particularly MMP-2 and MMP-9, which degrade the surrounding ECM [102,103]. Experimental data from co-culture models of PVAT and VSMCs demonstrated that exposure to inflamed PVAT results in increased VSMC proliferation and the expression of contractile markers, such as α-SMA and smooth muscle 22 alpha (SM22α), thereby contributing to vascular remodeling [102,104].

A reduction in adiponectin levels and an increase in TNF-α within PVAT contribute to the pathological development of neointimal hyperplasia after endovascular injury. In obesity, there is a decrease in beneficial adipokines and an increase in pro-inflammatory factors, such as TNF-α, both of which promote the proliferation of VSMCs and inflammatory responses within PVAT [101].

In summary, PVAT plays a dynamic and intricate role in vascular physiology and pathology, affecting VSMC growth and migration through the secretion of specific factors.

#### 4.3.3. Immune Cell Recruitment and Local Inflammation

Atherosclerosis is influenced not only by inflammation within the vascular intima (“inside-out”), but also by inflammatory signals originating from the PVAT (“outside-in”). These signals are crucial for the recruitment of inflammatory cells and disease progression, especially in the context of obesity [105].

**Figure 5:**
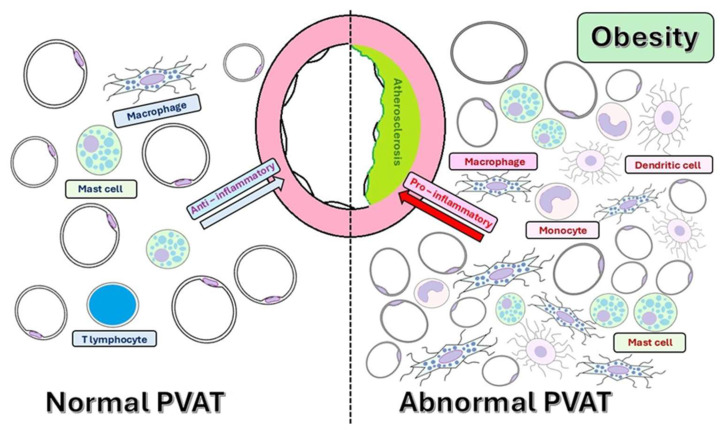
Cellular composition in normal vs. obese PVAT.

PVAT comprises a diverse array of cell types, including endothelial cells, macrophages, lymphocytes, lymphoblasts, pre-adipocytes, mature adipocytes, and mesenchymal stem cells. The proportions of these cell types can vary based on factors such as age, environmental conditions, and nutritional status. In obesity, there is a notable increase in the percentage of inflammatory cells in the periaortic PVAT, and metabolic stimuli significantly influence both the degree and extent of these changes [101]. Immune cells infiltrating the PVAT in obesity promote endothelial dysfunction, vasoconstriction, and VSMC proliferation. Consequently, leptin stimulates macrophages to release inflammatory cytokines, such as TNF-α and IFN-γ. Adipocytes also release C-C motif chemokine ligand 5 (CCL5) (also known as RANTES (regulated upon activation, normal T-cell expressed and secreted)), which recruits CD3+ T lymphocytes to the PVAT, resulting in heightened inflammation (Figure 5) [105].

The chemokines CCL2 (also known as MCP-1 (monocyte chemoattractant protein-1)), CCL5, and CXCL10 (also known as IP-10 (interferon gamma-induced protein 10)) play a central role in the initiation and progression of atherosclerosis by recruiting and activating inflammatory cells. Research using murine models of PVAT transplantation has shown an increase in the production of PVAT-derived CCL2, which subsequently contributes to the development of atherosclerosis. In humans, dysfunctional PVAT secretes adipokines and inflammatory cytokines, such as TNF-α, IL-6, and leptin. These substances, in turn, stimulate the production of endothelial CCL2, ICAM-1, and VCAM-1, which exacerbate endothelial dysfunction. This cascade of events leads to the recruitment of monocytes toward the inflamed endothelium, facilitating their infiltration into the vascular wall and contributing to the formation and progression of vascular lesions and the development of plaques [106].

CCL5, released by platelets and deposited on activated endothelium, interacts with the C-C chemokine receptor type 1 (CCR1) and CCR5 to induce leukocyte adhesion and transmigration, particularly of monocytes and neutrophils, into the intima. CXCL10, an IFN-γ-induced chemokine, is produced by VSMCs and acts through C-X-C chemokine receptor 3 (CXCR3) (), inhibiting endothelial healing and promoting leukocyte chemotaxis, as well as the proliferation and migration of endothelial and smooth muscle cells. Studies in murine models have shown that the absence of these chemokines or their receptors significantly reduces Th1 (CD4^+^ T-cell) accumulation, leukocyte infiltration, and atherosclerotic lesion size, confirming the pro-atherogenic role of these inflammatory mediators [106]. These immune cells, once recruited, become activated within PVAT or intimal spaces and release additional pro-inflammatory mediators, such as IFN-γ, IL-1β, and TNF-α, creating a self-amplifying loop of inflammation. Furthermore, macrophages in PVAT in pathological conditions demonstrate increased M1 polarization, while the proportion of regulatory T-cells decreases, exacerbating the inflammatory milieu [107].

#### 4.3.4. ECM Remodeling and Fibrosis

One of the most significant pathological processes that occurs in adipose tissue during obesity is fibrosis, which is characterized by excessive accumulation of ECM proteins, loss of tissue plasticity, and the development of chronic, low-grade inflammation [108]. The ECM in adipose tissue comprises collagens (types I, III, V, and VI), fibronectin, elastin, hyaluronic acid (HA), non-collagenous proteins such as secreted protein, acidic and rich in cysteine (SPARC) and thrombospondin-1 (TSP-1), as well as proteoglycans and glycoproteins [109,110]. During overnutrition, ECM accumulates and becomes rigid, contributing to the loss of adipocyte function, inflammation, and insulin resistance. Collagen VI plays a central role in fibrosis, especially the α3 subunit, from which endotrophin, a fragment with pro-fibrotic and pro-inflammatory effects, is derived [111,112].

As adipose tissue expands, angiogenesis may not keep pace with the increase in tissue volume, resulting in local hypoxia. This triggers the activation of hypoxia-inducible transcription factors, especially HIF1α, which acts as a master regulator of the pathological microenvironment [110,113]. HIF1α promotes the expression of lysyl oxidase (LOX, the enzyme that catalyzes covalent bonds in collagen and elastin fibers), MMP-14 (which degrades collagen VI and releases endotrophin), and collagen types I and III, thereby supporting the remodeling of the ECM and promoting fibrosis [110].

Interestingly, in obese adipose tissue, HIF1α does not promote vascular endothelial growth factor A (VEGF-A) and angiogenesis but instead exclusively enhances fibrogenesis, accentuating hypoxia and tissue dysfunction [110,114].

ECM stiffening and increased mechanical tension on adipocytes activate pro-inflammatory pathways, such as RhoA (Ras homolog gene family, member A (involved in vessel permeability and pro-inflammation)) and NF-κB, promoting the infiltration of pro-inflammatory M1 macrophages, CD8^+^ T and B lymphocytes, mast cells, and neutrophils [110,115,116]. This chronic low-grade inflammation contributes to insulin resistance and the deterioration of systemic metabolic function [117].

Inflammation may precede fibrotic development through the secretion of cytokines, such as IL-6 and TNFα, and the activation of transforming growth factor beta (TGF-β) pathways in adipocytes and stromal cells [118,119]. Consequently, fibrosis and inflammation amplify each other, creating a pathological vicious cycle in obese adipose tissue.

**Figure 6:**
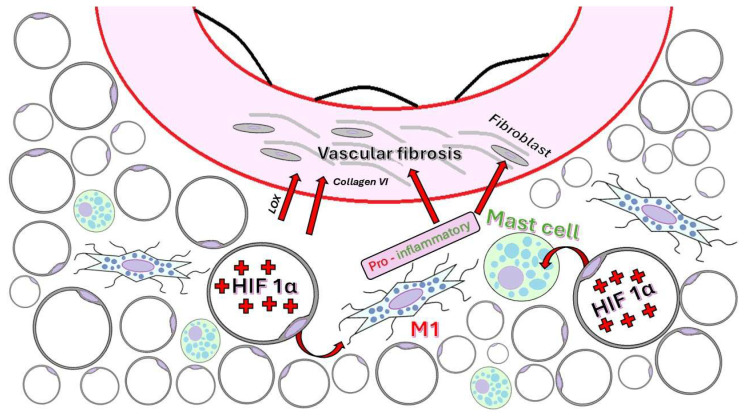
PVAT-induced vascular fibrosis.

The primary cellular components involved in fibrosis are:

(1) Adipocytes, which become a source of collagen VI and LOX via the action of HIF1α [112,120].
(2) Endothelial cells line the large macrovasculature within adipose tissue and contribute to vascular remodeling and fibrosis through angiocrine signaling and their potential to undergo endothelial–mesenchymal transition [121,122].
(3) M2 macrophages stimulate fibrogenesis via TGF-β by regulating the production of collagens [123], whereas M1 macrophages enhance inflammation, which exacerbates fibrosis [124,125].
(4) Adipose-derived stem cells and fibroblasts serve as significant sources of ECM when platelet-derived growth factor receptor alpha (PDGFRα) and CD9 are present [126,127].
(5) Mast cells are plentiful in obese adipose tissue and, through degranulation, release pro-inflammatory factors and fibrogenic mediators, such as CCL2 and histamine (Figure 6) [128,129].

PVAT is a rich source of enzymes and profibrotic mediators that influence the ECM composition of both PVAT and the adjacent vascular wall. In particular, MMPs, especially MMP-2 and MMP-9 (members of the gelatinase subgroup of matrix metalloproteinases), play a crucial role in ECM remodeling by degrading key structural components, such as type IV and V collagen, fibronectin, laminin, and elastin. These components are secreted by PVAT macrophages and pre-adipocytes, contributing to ECM degradation, smooth muscle migration, and plaque instability [130,131]. The increased expression of CCL2 and CCL5 in PVAT promotes perivascular fibrosis and accelerates vascular aging [132]. In periplaque PVAT, M2 macrophages have been linked to increased calcification, whereas M1 macrophages correlate with enhanced collagen deposition, highlighting their distinct contributions to vascular remodeling [133].

## 5. Pathological Features of PVAT in Human Atherosclerosis

Histopathological analyses of human vascular specimens obtained from autopsies or carotid endarterectomy procedures consistently demonstrate that PVAT adjacent to atherosclerotic plaques shows significantly more inflammation than PVAT surrounding non-diseased vessels. Microscopic evaluation using hematoxylin and eosin and Masson’s trichrome staining reveals substantial infiltration of inflammatory cells, particularly macrophages and lymphocytes, in PVAT near advanced lesions, especially in areas close to vulnerable or ruptured plaques [134]. Complementary immunohistochemistry studies further confirm the presence of an activated immune microenvironment within PVAT, demonstrating elevated expression of CD68 (macrophages), CD3 (T lymphocytes), and key pro-inflammatory cytokines, including TNF-α, IL-6, and CCL2 [135]. These molecular markers correlate with systemic inflammation and cardiovascular risk, suggesting that localized immune activation contributes to plaque progression. Notably, inflamed PVAT has been linked to features of plaque vulnerability, such as thin-cap fibroatheromas and necrotic core formation, with histological sections displaying increased MMP expression in areas where PVAT overlaps plaques with large lipid cores and reduced fibrous caps. Collectively, these findings emphasize the active and potentially pathogenic role of PVAT in destabilizing atherosclerotic plaques [135,136].

Morphometric analyses of PVAT reveal that adipocyte hypertrophy in perivascular adipose tissue is strongly correlated with adverse metabolic and cardiovascular risk factors, including higher triglycerides, remnant cholesterol, BMI, waist circumference, plasma glucose, homeostasis model assessment for insulin resistance (HOMA-IR), and visceral adiposity, as well as lower high-density lipoprotein and basal metabolic rate. In particular, enlarged PVAT adipocytes are associated with increased CD68^+^ macrophage infiltration, higher levels of pro-inflammatory cytokines, such as high-sensitivity CRP, TNF-α, and E-selectin (endothelial adhesion), and a greater proportion of metabolically active pro-inflammatory macrophages. These findings suggest that adipocyte enlargement in PVAT may act as an early indicator of vascular inflammation and metabolic dysfunction, even among otherwise healthy individuals [136].

These changes indicate adipocyte stress and dysfunction and are linked to local hypoxia, oxidative stress, and profibrotic signaling. Such morphological changes are early indicators of vascular risk and can be measured using both histology and advanced imaging techniques [137].

## 6. Human vs. Animal Model Findings

Comparative histological analyses have shown that commonly used atherosclerotic murine models, particularly ApoE^−^/^−^ (Apolipoprotein E knockout mice) and LDLR^−^/^−^ (low-density lipoprotein receptor knockout mice) mice, closely replicate many inflammatory features observed in human PVAT. In both of ApoE^−^/^−^ and LDLR^−^/^−^ models, PVAT adjacent to atherosclerotic arteries exhibits significant immune cell infiltration, with an increased presence of macrophages, lymphocytes, and neutrophils. These infiltrates are accompanied by the increased expression of pro-inflammatory cytokines, such as CCL2, TNF-α, and IL-8, reflecting a robust inflammatory environment. Additionally, substantial ECM remodeling, including fibrosis, has been reported in both human and murine PVAT surrounding diseased vessels. These findings reinforce the translational value of ApoE^−^/^−^ and LDLR^−^/^−^ models in mimicking human PVAT pathology, highlighting their utility in studying the role of PVAT inflammation and structural remodeling in the progression of atherosclerosis. While the anatomy and adipocyte composition of PVAT differ between humans and rodents, the cellular and molecular pathways involved in PVAT-driven inflammation appear to be conserved, which supports the use of animal models for mechanistic studies and therapeutic testing [138,139].

## 7. Imaging Modalities for the Evaluation of PVAT

Computed tomography (CT) utilizing the fat attenuation index (FAI) has become a crucial imaging technique for quantifying PVAT inflammation. FAI assesses the radiodensity of adipose tissue adjacent to coronary arteries; inflamed PVAT shows higher attenuation values because of increased cellularity, fibrosis, and edema [140]. A higher FAI in vessels with obstructive coronary artery disease underscores the relationship between PVAT inflammation and atherosclerotic processes rather than acute vasospastic events [141]. FAI changes are associated with the burden of coronary artery disease and independently predict future cardiovascular events, regardless of traditional risk factors [142].

Magnetic resonance imaging (MRI) provides high-resolution soft-tissue contrast, enabling the assessment of PVAT volume and composition. Although MRI is less commonly used in clinical settings for PVAT, it can detect changes in adipocyte structure and perivascular fibrosis in both humans and animal models [143].

Positron emission tomography (PET), especially when using tracers like Fluorine-18 Fluorodeoxyglucose (18F-FDG), can detect metabolic activity in PVAT, which reflects inflammation. Enhanced 18F-FDG uptake in PVAT is associated with systemic inflammatory states and vulnerable plaques [144].

Clinical imaging modalities, such as PET-CT and CT-derived FAI, have shown that higher BMI correlates with increased PVAT volume and inflammatory activity, independent of plaque burden [145].

Coronary inflammation causes dynamic changes in the water–lipid balance in PVAT, as reflected by the perivascular FAI on coronary computed tomography angiography (CCTA). A group of authors hypothesized that other radiomic signatures associated with fibrosis and microvascular remodeling might also contribute to predicting cardiac risk. They developed a new artificial intelligence (AI)-based method for analyzing the radiomic profile of coronary PVAT, which was validated in three studies [146]. In their first study, FAI was associated with inflammation and radiomic texture with fibrosis and vascularization. In their second study, the radiomic features of PVAT were analyzed, and a machine learning algorithm (FRP—fat radiomic profile) was trained that efficiently predicted cardiac risk. In their third study, 44 patients with acute myocardial infarction were evaluated, and FRP was significantly higher than in controls and remained unchanged at 6 months, unlike FAI. This result shows that FRP captures persistent structural changes in PVAT, not just acute inflammation. The authors concluded that radiomic profiling of coronary PVAT using CCTA and AI enables the detection of perivascular structural remodeling associated with coronary artery disease, and the new AI biomarker, FRP, offers a significant improvement in predicting cardiac risk [146].

In addition to imaging, circulating biomarkers can provide indirect evidence of PVAT dysfunction and inflammation. High-sensitivity CRP serves as a general marker of systemic inflammation and has been shown to correlate with FAI measurements and PET-derived metabolic activity in PVAT. Although not specific to PVAT, elevated high-sensitivity CRP supports the existence of a systemic inflammatory environment that involves dysfunctional perivascular fat [147]. Combining imaging with circulating biomarkers enables a more comprehensive risk stratification and may guide personalized therapeutic strategies aimed at targeting PVAT inflammation [148].

## 8. Emerging Biomarkers of PVAT Dysfunction

Galectin-3 (Gal-3) is a protein that regulates essential cellular functions, including growth, differentiation, and proliferation. It plays a key role in biological processes, including chronic inflammation and adipose tissue and ECM remodeling, as well as the modulation of macrophage chemotaxis and phagocytosis [149]. Gal-3 is a β-galactosidase-binding lectin that has a C-terminal region with a carbohydrate recognition domain (CRD) and an N-terminal region rich in proline-glycine-alanine-tyrosine repeats. This structure endows it with a dual role in inflammation, displaying both pro-inflammatory and anti-inflammatory properties [150].

Gal-3 can bind to advanced glycation end products (AGEs) and advanced lipoxidation end products (ALEs), which accumulate in target organelles, exerting toxic effects and triggering pro-inflammatory and pro-oxidant pathways. Therefore, Gal-3 is associated with a protective effect against the development of renal diseases and atherosclerosis in experimental animals. In the absence of Gal-3, the AGE receptor (RAGE), which mediates tissue damage induced by AGEs and ALEs, is upregulated [151,152].

Gal-3 is expressed in adipose tissue by adipocytes and infiltrating macrophages, with circulating levels elevated in both obese humans and obese experimental animals [150,153]. Its production is associated with higher levels of visceral fat, which mediate inflammation and fibrosis in cardiovascular tissue, promoting the development of atherosclerosis. Gal-3 contributes to the promotion of pre-adipocyte differentiation in vitro, indicating its role in the expansion of adipose tissue during obesity [154]. This is supported by an experimental study involving male Wistar rats that were fed either a high-fat diet (33.5% fat) or a standard diet (3.5% fat) for 6 weeks. The obese animals exhibited elevated levels of Gal-3, which were associated with increased pericellular collagen, heightened tissue inflammation, and improved differentiation of adipocytes [155].

Gal-3 plays a role in initiating and sustaining perivascular inflammation, which promotes the progression of cardiovascular diseases. It has been studied for its potential to predict cardiovascular risk in systemic inflammatory conditions, such as rheumatoid arthritis [156].

In diabetic and obese individuals, Gal-3 has been observed to evolve alongside impaired glucose homeostasis. However, other experimental studies conducted on Gal-3-deficient mice fed a high-fat diet demonstrated increased adiposity and systemic inflammation, along with the emergence of altered glucose homeostasis [151].

Therefore, these findings suggest that Gal-3 requires further investigation to clarify and modulate its role as a contributor or therapeutic target in obesity and type 2 diabetes. The inhibition of Gal-3 through the administration of modified citrus pectin (MCP) has been experimentally shown to reduce inflammation, fibrosis, and pathological tissue remodeling [155]. Its potential as a biomarker in PVAT may be important for the early detection of cardiovascular diseases [156].

Epigenetic alterations, particularly due to the altered expression of specific miRNAs (miRs), are implicated in PVAT dysfunction. These post-transcriptional regulators affect the expression of genes related to inflammation, cell migration, angiogenesis, and vascular homeostasis. Several miRNAs have received attention in the recent literature [157].

Brown adipocytes act as the primary site for energy expenditure and heat production, existing not only in classical BAT but also in WAT. Blocking miR-155 is associated with enhanced differentiation of brown adipocytes, while in white adipocytes, blocking miR-155 is associated with the induction of a brown-adipocyte-like phenotype. In transgenic mice, the overexpression of miR-155 leads to reduced brown tissue mass and altered functionality [158].

TNF-α treatment of human adipocytes, along with studies involving mice, results in a two- to sixfold increase in miR-155 expression within 3 to 24 h. TNF-α dose-dependently upregulates miR-155 in primary cultures of human adipocytes. The activation of NF-kB mediates this effect and inhibiting NF-kB lowers miR-155 expression. Transgenic models with activation or suppression of p65 in adipose tissue show a corresponding increase or decrease in miR-155 [159].

Karkeni et al. demonstrated differences in miR-155 expression in adipose tissue biopsies from lean and obese subjects, with higher expression in obese patients, and positive correlations with BMI and TNF-α expression [159]. Overfed mice exhibit a reduction in brown adipose tissue mass, along with impaired thermogenic function. Similarly, transgenic overexpression of miR-155 results in a decrease in brown tissue mass and function, while the opposite is observed in miR-155-deficient mice [158]. The increased expression of miR-155 in adipocytes likely creates an inflammatory loop, possibly through the effects of PPARγ and the transcription factor C/EBPβ [158,159].

Antagonizing miR-155 by administering antagomir negatively impacts TNF-α and IL-6, indicating therapeutic potential by reducing the expression of adipogenic and lipogenic markers [159,160]. Considering the established role of miR-155 in inflammatory processes affecting adipocytes, it is likely that it also influences PVAT, thereby impacting vascular function.

The miR-126 precursor is primarily expressed in endothelial cells and produces two mature miRNAs, miR-126-3p and miR-126-5p, which regulate cell junction integrity, angiogenesis, and the suppression of the inflammatory response. miR-126-5p enhances endothelial regeneration after denudation, and its expression is reduced in areas susceptible to atherosclerosis [161]. MiR-126-3p enhances VEGF signaling during angiogenesis and supports an endothelial phenotype characterized by reduced inflammatory activation. It achieves this by inhibiting adhesion molecules, such as VCAM-1 and ICAM-1, decreasing monocyte infiltration, and limiting the progression of the atherosclerotic process [161]. If PVAT is healthy, miR-126 indirectly maintains an anti-inflammatory profile by regulating the functions of endothelial cells and endothelial progenitor cells (EPCs). In conditions of ischemia and vascular inflammation, the expression of miR-126 increases in EPCs, stimulating their proliferation and migration while reducing EPC apoptosis. This positively impacts vascular repair processes by enhancing VEGF-dependent stimulation, promoting angiogenesis and endothelial regeneration via the Notch pathway, and inhibiting vascular inflammation through the repression of specific pro-inflammatory factors, such as VCAM1 [162,163]. On the other hand, reduced miR-126 expression in dysfunctional PVAT may be associated with the loss of anti-contractile effects and the initiation of pro-atherogenic conditions.

## 9. Challenges, Limitations, and Future Directions

Despite promising results, specifically targeting PVAT remains challenging. Most therapies produce systemic effects, making it difficult to isolate PVAT-specific mechanisms. Additionally, PVAT is anatomically heterogeneous, and therapeutic responses may vary by location, e.g., coronary vs. aortic PVAT [44,164].

Imaging techniques such as CT-derived FAI and PET-CT may aid in monitoring regional responses to therapy, but more targeted delivery systems or PVAT-specific biomarkers are necessary. Future research should investigate nanoparticle-based delivery or local modulation strategies to minimize systemic side effects [165,166].

Another limitation is the lack of standardized methods to assess PVAT inflammation across studies, which makes comparisons and clinical translation challenging. Developing unified criteria for PVAT assessment will enhance therapeutic development.

Lifestyle modifications are essential for restoring PVAT homeostasis. Weight loss, achieved through caloric restriction or bariatric surgery, results in a reduction in PVAT volume and inflammation, a decrease in adipocyte size, and an increase in adiponectin secretion, shifting the PVAT phenotype toward an anti-inflammatory state [167]. In obese mice, reduced adiponectin expression in PVAT is associated with impaired endothelium-dependent relaxation, decreased NO availability, and increased oxidative stress. Aerobic exercise restored adiponectin receptor expression in PVAT, enhancing vascular function by normalizing NO production and decreasing reactive oxygen species [168].

Physical activity reduces PVAT inflammation by improving insulin sensitivity and balancing adipokine levels. Exercise training has been shown to decrease macrophage content and inflammatory gene expression in PVAT of both humans and rodents [169].

Dietary patterns, particularly plant-based and low-carbohydrate diets, have a positive influence on PVAT biology by modulating systemic metabolism and reducing oxidative stress. Specific nutrients, such as omega-3 fatty acids and polyphenols, are associated with a reduction in perivascular fat inflammation [170].

Further research is needed to understand the epigenetic mechanisms linking inflammation, oxidative stress, and vascular dysfunction in the PVAT of obese patients.

## Figures and Tables

**Table 1:** Main adipokines.

Adipokine	First Mentioned	First Described in Connection with PVAT	Functional Role in PVAT Under Physiological Conditions
Adiponectin	1996—was mentioned as apM1 [35] 1999—mentioned as adiponectin [36]	2007 [37]	Enhances endothelial function and increases NO bioavailability
Leptin	1994—was mentioned as ob gene [38] 1995—was mentioned as leptin [39]	2000 [40]	Promotes neovascularization and enhances endothelial function
Omentin	2005 [41]—was mentioned in an article published in PubMed	2012 [42]	Exerts anti-inflammatory and anti-atherosclerotic effects, enhances vascular tone

**Table 2:** Features of subcutaneous adipose tissue, visceral adipose tissue, and perivascular adipose tissue.

Feature	SAT	VAT	PVAT
Adipocyte size [45,50]	Small	Large	Heterogeneous (varies by the surrounding vessel)
Adipokines [45,51]	↑ Resistin, ↑ leptin, and no difference in adiponectin	↑ IL-12 IL-13, TNF-α, IL-6, IL-8, MCP-1, PAI-1, TBX2, PGE2; ↓ resistin, leptin ↓, adiponectin	Variable Secretion: Includes adiponectin, leptin, IL-6, TNF-α, context- and location-dependent
Immune cell infiltration [45,52]	Lower	Higher (especially macrophages and T-cells)	Mixed (either anti-inflammatory or pro-inflammatory)
Types of adipose tissue [45,53]	White adipose tissue	White adipose tissue	BAT-like (thoracic), WAT-like (abdominal)
Embryological origin [43,45,53,54]	Mesoderm: Derived from Wt1-negative precursor cells	Mesoderm: Partially derived from Wt1-positive precursor cells	Mixed (mesoderm + neural crest in some regions). Heterogeneous embryonic origin is shown by different precursors depending on the region, such as SM22α+, Myf5+, Wt1+, or Pdgfra+ cells.
Clinical relevance [45,55]	Indicative of increased cardiovascular and metabolic risk	May have a relatively protective role	Depends on positioning whether it has a cardiovascular protective or proatherogenic role
Vascularization [45,54,56]	Moderate capillary supply	Rich vascular supply	Directly connected to the vessel vasculature
Innervation [45,56]	Low	Moderate	High (dense sympathetic nerve supply)
UCP1 expression/browning [53,57]	Absent	Absent	High in the thoracic region (UCP1+) and absent in other areas
Insulin sensitivity [45,58]	High	Low	Variable (dependent on localization)
CVD association/atherosclerosis risk [10,59]	Low association	High risk predictor	Independent predictor for CVD/atherosclerosis
Phenotypic plasticity [14,54]	Low	Limited	High

**Table 3:** PVAT-derived inflammatory markers and endothelial dysfunction.

Inflammatory Marker	Main Source in PVAT	Effects on Blood Vessels	Study Type	Number of Subjects
Leptin	Adipocytes	Stimulates production of TNF-α and IL-6; promotes vascular inflammation and atherosclerosis	Cross-sectional observational study [76]	125 patients with coronary artery disease (CAD) who needed direct myocardial revascularization through coronary artery bypass graft (CABG) surgery
TNF-α	Macrophages, adipocytes	Induces expression of ICAM-1 and VCAM-1; promotes leukocyte infiltration and endothelial dysfunction	Cross-sectional observational study [77]	32 patients with known CAD who underwent CABG surgery
IL-6	Macrophages, adipocytes	Promotes arterial stiffness and endothelial dysfunction via inflammation and extracellular matrix remodeling mechanisms	Experimental study [78]	8 low-density lipoprotein receptor (LDLr)-deficient mice (LDLr^−^/^−^)
Resistin	Macrophages	PVAT-derived resistin disrupts endothelial potassium channel–mediated vasorelaxation in hypertensive rats	Experimental study [79] (SHRSP rats)	5 rats per sex/group
Visfatin/eNampt	Adipocytes	Impairment of endothelium-dependent vasodilation and increased oxidative stress through pro-inflammatory signaling	Original experimental studies [80]	6 C57BL/6 mice per group
Endothelin-1 (ET-1)	Endothelial cells, adipocytes	Plays a crucial role in obesity-induced PVAT dysfunction by inhibiting Nrf2 and elevating oxidative stress	Experimental study performed on animal models (mice) [81]	55 male C57BL/6J mice
IL-18	Adipocytes, macrophages	Inhibits eNOS through the Tak1 pathway, impairing endothelium-dependent relaxation	Experimental study [82]	8 high-fat diet-fed mice and adipocyte cultures
Chemokine (C-C motif) ligand 2 (CCL2) (MCP-1)	Adipocytes, macrophages	Promotes monocyte infiltration and endothelial dysfunction	Diet-induced obese mice, interventional study [83]	10–12 mice/group
IL-17	T helper 17 lymphocytes	Causes oxidative stress, vascular fibrosis, and impairs vasorelaxation	In vivo/ex vivo transgenic mouse study [84]	8 mice/group

## Data Availability

Data are available upon request from the corresponding author.

## Abbreviations

AGEs	Advanced Glycation End Products
ALEs	Advanced Lipoxidation End Products
AI	Artificial Intelligence
BAT	Brown Adipose Tissue
BMI	Body Mass Index
CCTA	Coronary Computed Tomography Angiography
CCL5	C-C Motif Chemokine Ligand 5
CCL2	C-C Motif Chemokine Ligand 2
CXCL10	C-X-C Motif Chemokine Ligand 10
CXCR3	C-X-C Chemokine Receptor 3
CRD	Carbohydrate Recognition Domain
CRP	C-Reactive Protein
CT	Computed Tomography
EAT	Epicardial Adipose Tissue
ECM	Extracellular Matrix
EPC	Endothelial Progenitor Cells
ERK	Extracellular Signal-Regulated Kinase
FABP	Fatty Acid-Binding Protein
FAI	Fat Attenuation Index
FDG	Fludeoxyglucose
FRP	Fat Radiomic Profile
HDL	High-Density Lipoprotein
HOMA-IR	Homeostasis Model Assessment For Insulin Resistance
ICAM-1	Intercellular Adhesion Molecule-1
IFN-γ	Interferon Gamma
IL-17A	Interleukin-17a
IL-18	Interleukin-18
IL-1β	Interleukin-1 Beta
IL-6	Interleukin-6
IL-8	Interleukin-8
IP-10	Interferon Gamma-Induced Protein 10
LAD	Left Anterior Descending Artery
LCN-2	Lipocalin-2
LDL	Low-Density Lipoprotein
LDLR	Low-Density Lipoprotein Receptor
LOX	Lysyl Oxidase
MAPK	Mitogen-Activated Protein Kinase
MCP	Modified Citrus Pectin
MCP-1	Monocyte Chemoattractant Protein-1
MMPs	Matrix Metalloproteinases
MMP-2	Matrix Metalloproteinase-2
MMP-9	Matrix Metalloproteinase-9
MRI	Magnetic Resonance Imaging
NADPH	Nicotinamide Adenine Dinucleotide Phosphate
NF-kB	Nuclear Factor Kappa-Light-Chain-Enhancer of Activated B Cells
NLRP3	Nod-, Lrr-, And Pyrin Domain-Containing Protein 3
NO	Nitric Oxide
PET	Positron Emission Tomography
PET-CT	Positron Emission Tomography–Computed Tomography
PGC-1α	Peroxisome Proliferator-Activated Receptor Gamma Coactivator 1-Alpha
PKC-β	Protein Kinase C Beta
PVAT	Perivascular Adipose Tissue
RAGE	Receptor for Advanced Glycation End Products
RANTES	Regulated Upon Activation, Normal T-Cell Expressed and Secreted
ROS	Reactive Oxygen Species
SAT	Subcutaneous Adipose Tissue
α-SMA	Alpha-Smooth Muscle Actin
SM22α	Smooth Muscle 22 A
SPARC	Secreted Protein, Acidic and Rich in Cysteine
TGF-β	Transforming Growth Factor Beta
TNF-α	Tumor Necrosis Factor-Alpha
TSP-1	Thrombospondin-1
UCP-1	Uncoupling Protein-1
VAT	Visceral Adipose Tissue
VCAM-1	Vascular Cell Adhesion Molecule-1
VEGF-A	Vascular Endothelial Growth Factor
VSMC	Vascular Smooth Muscle Cell
WAT	White Adipose Tissue
